# Parametric equations to study and predict lower-limb joint kinematics and kinetics during human walking and slow running on slopes

**DOI:** 10.1371/journal.pone.0269061

**Published:** 2022-08-04

**Authors:** Anat Shkedy Rabani, Sarai Mizrachi, Gregory S. Sawicki, Raziel Riemer

**Affiliations:** 1 Department of Industrial Engineering & Management, Ben-Gurion University of the Negev, Beersheba, Israel; 2 School of Mechanical Engineering and the School of Biological Sciences, Georgia Tech, Atlanta, GA, United States of America; University of Pittsburgh, UNITED STATES

## Abstract

Comprehensive data sets for lower-limb kinematics and kinetics during slope walking and running are important for understanding human locomotion neuromechanics and energetics and may aid the design of wearable robots (e.g., exoskeletons and prostheses). Yet, this information is difficult to obtain and requires expensive experiments with human participants in a gait laboratory. This study thus presents an empirical mathematical model that predicts lower-limb joint kinematics and kinetics during human walking and running as a function of surface gradient and stride cycle percentage. In total, 9 males and 7 females (age: 24.56 ± 3.16 years) walked at a speed of 1.25 m/s at five surface gradients (-15%, -10%, 0%, +10%, +15%) and ran at a speed of 2.25 m/s at five different surface gradients (-10%, -5%, 0%, +5%, +10%). Joint kinematics and kinetics were calculated at each surface gradient. We then used a Fourier series to generate prediction equations for each speed’s slope (3 joints x 5 surface gradients x [angle, moment, mechanical power]), where the input was the percentage in the stride cycle. Next, we modeled the change in value of each Fourier series’ coefficients as a function of the surface gradient using polynomial regression. This enabled us to model lower-limb joint angle, moment, and power as functions of the slope and as stride cycle percentages. The average adjusted R^2^ for kinematic and kinetic equations was 0.92 ± 0.18. Lastly, we demonstrated how these equations could be used to generate secondary gait parameters (e.g., joint work) as a function of surface gradients. These equations could be used, for instance, in the design of exoskeletons for walking and running on slopes to produce trajectories for exoskeleton controllers or for educational purposes in gait studies.

## 1. Introduction

Kinematic and kinetic data describing the dynamics of lower-limb joints are critical for understanding the neuromechanics and energetics of human locomotion [1] and guiding the design of assistive devices (e.g., lower-limb exoskeletons). From a basic science perspective, studies have provided insights into the sources of metabolic energy consumption by relating joint kinetics to whole-body oxygen consumption [2,3]. From a more specific applied science perspective, kinematic and kinetic data are important for sizing the geometry and material properties of lower-limb orthoses [4,5] and motors for powered exoskeletons and prostheses [6,7], as well as for guiding control software in both powered exoskeletons [8] and controllers for commercial devices, such as ATLAS [9], ReWalk [10], and eLegs [11], and prostheses [12,13].

Combining experimental approaches like motion capture and force measurements with rigid body models (i.e., inverse dynamics) provides means for obtaining lower-limb joint kinematics and kinetics data. Accordingly, many studies have already addressed walking or running at typical speeds on level ground (e.g., [2,14,15]). However, recently, several papers have instead focused on the kinematics and kinetics of slope walking (e.g., [16–19]) and slope running (e.g., [9,16,20–22]); however, only one study [19] analyzed the 3D joint during walking. Moreover, to the best of our knowledge, joint kinematics and kinetics of slope running on three anatomical planes has only been reported on moderate slopes of up to 7% [22]. As such, our study provides a wider range of surface gradients compared to the current literature. Sloped surface walking and running is of particular interest when designing lower-limb exoskeletons and prostheses to augment, restore, or harvest joint-level mechanical gait energy because in real life applications, the assistive device user does not only walk or run on level surfaces.

Further, data acquisition of motion and force during locomotion is expensive and time consuming, meaning the available joint-level data is limited to only a small subset of speeds. The same holds for up- and downhill locomotion data, perhaps due to the added difficulty of instrumenting sloped surfaces. Additionally, relationships describing how lower-limb joint kinematics and kinetics depend on the gait phase have not been documented using parametric equations, which, if developed, can provide pertinent data for the design of assistive devices without requiring exhaustive data acquisition. Parametric equations enable kinetics and kinematics calculations on a range of slopes, not only the slopes measured in gait lab. These could be useful in many applications: (1) As a reference for an exoskeleton, a prosthesis, or a walking robot controller that needs to engage different slopes or change gait modes (without the need to model the raw data); (2) In device optimization where having an equation enables much simpler modeling of the optimization problem; (3) In education, enabling students to produce joint parameters of many gait states and analyze them, a task that would be much harder to perform using raw data.

Using an experimental data set, our study aimed to develop a comprehensive 3D parametric model of lower-extremity joint kinematics and kinetics during human walking and slow running up and down slopes. This describes the average lower-limb joint kinematics and kinetics of a healthy, relatively young (up to 40 years old) population.

## 2. Methods

### 2.1 Experimental protocol

We collected gait data in two separate experiments at two different labs (Ben-Gurion University (BGU) and North Carolina State University (NCSU)) from 16 healthy adults, with no lower-limb injuries or impairments: 9 males and 7 females (age: 24.56 ± 3.16 years, [Range 18–28 years]; height: 1.73 ± 0.09 m, [range: 1.55–1.86 m]; mass: 68.01 ± 13.98 kg, [range: 45.0–88.7 kg]). The height and mass ranges cover approximately 90% of the US and Israeli demographics [23,24]. All participants signed an informed consent form approved by either the University of North Carolina at Chapel Hill Human Research Institutional Review Board or the Ben-Gurion University of the Negev Human participants Research Committee. All participants walked on an instrumented split-belt treadmill (Bertec Inc., Columbus, OH, USA) at a speed of 1.25 m/s and at five different surface gradients (-15%, -10%, 0%, +10%, +15%), as well as ran at a speed of 2.25 m/s and at five different surface gradients (-10%, -5%, 0%, +5%, +10%). The participants from BGU also walked and ran on an additional four slopes for each speed (-12.5%, -5% +5%, +12.5% for walking; -7.5%, -2.5%, +2.5%, +7.5% for running). Motion and force data were collected at each surface gradient using a minimum of 7 gait cycles, with an average of 16 cycles per condition. Using this data for each participant, the average joint kinematics and kinetics were calculated.

### 2.2 Data acquisition

Motion data were collected using a motion caption system (eight-camera MX40+, Vicon Inc., Oxford, UK or fourteen-camera Oqus, Qualisys Medical AB®, Gothenburg, Sweden) at a frequency of 120 Hz to capture the positions of 22 reflective markers attached to each participant’s pelvis and right leg (modification of the Calibration Anatomical System Technique (CAST) marker set [25]; see **S1 Fig** for more information regarding marker placements). Clusters of three or four markers were placed on rigid plates and attached to the pelvis, thigh, and shank segments to record their movement while running and walking.

Ground reaction force data were recorded as the participants walked and ran using two force platforms embedded in the treadmill (Bertec Inc, Columbus, OH, USA) at a frequency of 120 Hz. To measure the forces acting on each foot, the participants were instructed to place each foot on the corresponding side force plate. Both the marker positions and ground reaction raw data were low-pass filtered (Butterworth second order forward and backward passes) with a cut-off frequency of 10 Hz for motion and 35 Hz for ground reaction force data.

### 2.3 Lower-limb joint kinematics and kinetics calculations

**Fig 1:**
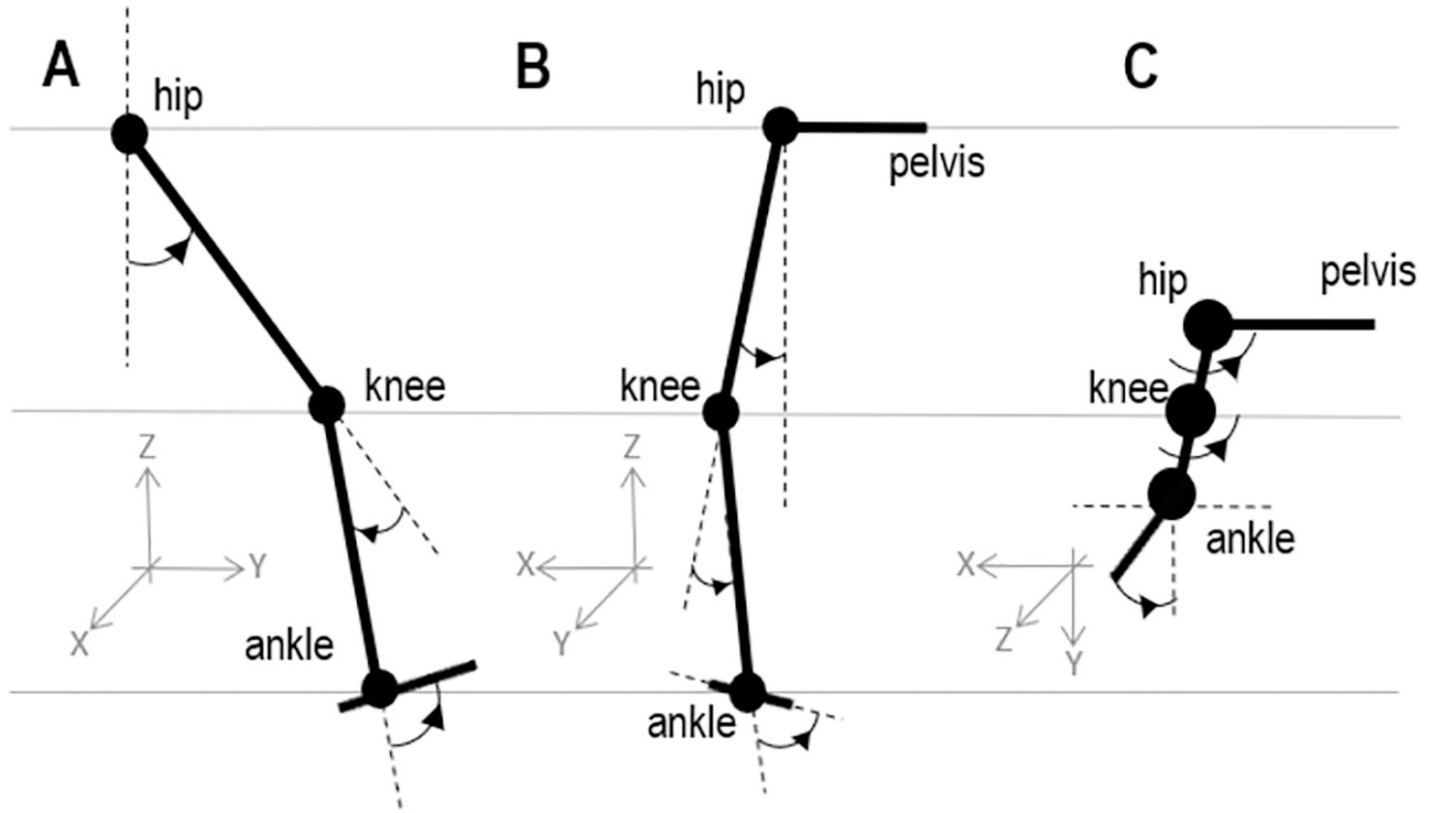
Definition of joint angles in three anatomical planes. For each angle, the arrow points in the direction of the positive angle (increase in angle value). Ankle angle was defined as the angle between the foot and shank segments; knee angle was calculated as the angle between the thigh and shank; and hip angle was calculated between the thigh and pelvis. A: Sagittal plane. In a neutral standing position when the foot is flat on the ground, ankle angle measures slightly less than 90°; knee angle measures 0° when the shank and thigh are in a perfectly straight line; and hip angle measures close to 0°. B: Frontal plane. C: Transverse plane. In both planes, frontal and transverse, ankle angle measures 0° when the foot and shank are in a perfectly straight line/the same plane, while knee and hip angles in these planes are 0° in a neutral anatomical standing posture.

Using Visual 3D (C-Motion Inc., Germantown, MD, USA) and assuming that both legs behaved symmetrically, ankle-, knee-, and hip-joint 3D kinematics and kinetics (joint angles and net joint movements) were calculated for the right leg only in three anatomical planes—sagittal, frontal, and transverse. We captured 5–20 s of static standing data to build a model for each participant’s specific body size (6 degrees of freedom). The markers’ positions on the segment endpoints in a static standing trial were used to calibrate a four-segment model (pelvis, thigh, shank, and foot) that calculated the body segment parameters (segment mass, center of mass, and moment of inertia) using the regression equations from [26]. Joint mechanical power was calculated for each joint by multiplying net joint moment by angular velocity. All calculated joint angles (defined in **Fig 1** and **S1 Table**), moments, and mechanical powers were normalized in time as percentages of one stride cycle using spline interpolation (MATLAB, MathWorks, Inc., Natick, MA). For each participant, the average joint kinematics and kinetics where calculated. Next moment and mechanical power time series were then normalized per each participant’s height and mass [27]. The coordinate system used a standard Cardin X-Y-Z sequence (X = medial/lateral axis, Y = anterior/posterior axis, Z = superior/inferior axis). Angular velocities were calculated using numerical differentiations of the joint angles (central difference). All participants’ experimental results were included and can be downloaded online (see S1 Data).

### 2.4 Modeling parametric equations to predict lower-limb joint dynamics for running on gradients

Our goal was to develop equations to predict the hip-, knee-, and ankle-joints’ angles, moments, and mechanical powers (9 total equations for each activity and plane of motion, parameterized by surface gradient and stride-cycle percentage). After calculating the stride-cycle average kinematics and kinetics for all available stride cycles and participants per each lower-limb joint (i.e., ankle, knee, and hip) across five surface gradients, we created training data by randomly selecting 14 participants (7 from BGU and 7 from NCSU); the remaining two participants (one from each lab) were later used for test data. Additionally, four slopes (-12.5%, -5% +5%, +12.5% for walking; -7.5%, -2.5%, +2.5%, +7.5% for running) that were not included in the model’s development were used to test the equations’ performance on data that wasn’t used to train the model.

The prediction equations were developed in two stages. In Stage 1, we used a Fourier series to develop prediction equations for each of the five training surface gradients, yielding a total of 45 equations for each plane of motion (3 lower-limb joints x 5 surface gradients x [angle, movement, mechanical power]) for each mode of locomotion (i.e., walking or running). For each joint and surface gradient, the equation input was the point in the stride cycle (i.e., stride cycle percentage). We defined a full stride cycle as heel strike to heel strike of the same limb, with the variable starting at 0% and ending at 100%. In Stage 2, we used Fourier series equations for each surface gradient and a regression analysis to develop a set of equations that predict a given stride average time series (e.g., lower-limb joint angle, moment, or mechanical power) for a given joint as a continuous function of (1) the surface gradient and (2) stride cycle percentage. Due to their continuity, these equations could predict a time series for a given joint even for surface gradients we did not measure (e.g., 2.5% grade). Hereafter, the term *lower-limb joint variable* refers to the time series data for the angle, moment, and mechanical power of each lower-limb joint, and the term *input parameter* refers to (1) the surface gradient during running or walking and (2) stride cycle percentage. To evaluate the prediction equations’ goodness-of-fit, we used adjusted R^2^ and root mean square error (RMSE) [28]. Additionally, the variability between the participant results were calculated in the form of participants standard deviation. This variability could be due to real individual differences and errors in the motion measurements (e.g., skin artifacts) or inverse dynamics calculations [29,30].

#### 2.4.1. Stage 1: Using Fourier series expansions to fit equations for joint kinematics and kinetics at each surface gradient

To develop an equation for each surface gradient as a function of stride cycle percentage, we tested several curve-fitting methods (e.g., n-polynomial, wavelet). Since locomotion is a periodic behavior, we decided to use a Fourier series in the form of sine and cosine trigonometric functions. To reduce the number of variables, we removed the phase shift terms in the arguments for sine and cosine (**Eq 1**). Next, to find the equation parameters (frequency (*ω*_i_) and coefficients (*β*_*o*_, *δ*_*i*_, *γ*_*i*_)), we performed a nonlinear optimization using R (http://www.r-project.org). Due to computational complexity, the optimization did not always converge, so we then transformed the optimization into a linear problem. From the nonlinear optimization results, we observed that the frequencies obtained in all surface gradients ranged from 0.03 to 0.07. Since the stride cycle is normalized to 100 points, we set the frequency value in each fit according to $2\pi f=2\pi \frac{1}{T}=2\pi \frac{1}{100}$. After determining frequency, the only unknown variables were the equation constant (*β*_*0*_) and the sine and cosine coefficients (δ_i_, γ_i_):

$$\beta_{0}+\sum_{i=1}^{n}\left[\delta_{i}\times \sin\left(i\omega x\right)+\gamma_{i}\times \cos\left(i\omega x\right)\right]$$ (1)

where *n* is the order of the series and *x* is time (stride cycle percentage). This formulation led to a linear optimization problem and guaranteed convergence to an optimal solution. However, we did not know a priori the order of the Fourier series required to represent the data well.

To obtain a first approximation of the minimal order required for the Fourier series, we calculated the signal power density for each lower-limb joint variable (e.g., angle). The main signal components were identified when the lowest included frequency was > 5% of the frequency with maximum amplitude. Examining the results of the five surface gradients for each participant and at each joint revealed that for a given lower-limb joint variable (e.g., moment), the number of components required (series order) was similar across all participants. Therefore, we used the average number of components across participants as the initial Fourier series size.

We then determined the final series size using the following four rules as guidelines. First, the series size for each lower-limb joint variable had to be the same across all surface gradients (e.g., representing ankle joint angle by a fifth order series for gradients from -10% to +10%). Second, the adjusted R^2^ of the fit had to be higher than 95%; if the initial size did not yield a sufficient R^2^, the series size was increased by a maximum order of two. Third, the series size was reduced to achieve a more compact equation for an R^2^ higher than 95%, with this reduction performed until the change in the average adjusted R^2^ was a maximum of 1% less than the initial R^2^. Fourth, insignificant coefficients (p < 0.05) on at least three of the five surface gradients were removed from the Fourier series. Using these rules, we obtained a total of 90 equations, with 45 separate Fourier series prediction equations corresponding to a stride time series for three lower-limb joint (ankle, knee, and hip) variables (angle, moment, and mechanical power) at each of the five training surface gradients at each speed (walking or running).

#### 2.4.2 Stage 2: Using regression equations to generalize the Fourier series equations to predict lower-limb joint kinematics and kinetics at any surface gradient

Our overall goal was to predict the lower-limb joint variables during human walking or running as a function of the following two variables: surface gradient and stride cycle percentage. To achieve this goal, we used polynomial regressions to model how the Fourier series coefficients *β*_*o*_, *δ*_*i*_, *γ*_*i*_ in **Eq 1** change as a function of surface gradient while running, yielding **Eqs 2–4**:

$${\beta_{0}=\sigma_{0}+\sum_{j=1}^{m}\sigma_{j}\times Surface\;Gradient}^{j}$$ (2)

$$\delta_{i}=\rho_{i0}+\sum_{j=1}^{m}\rho_{ij}\times {Surface\;Gradient}^{j}$$ (3)

$$\gamma_{i}=\mu_{i0}+\sum_{j=1}^{m}\mu_{ij}\times {Surface\;Gradient}^{j}$$ (4)

where *i* is the index for the coefficients from **Eq 1**; *j* is the index of the polynomial component; *m* is the polynomial order; and Surface Gradient is the vertical increase in height divided by the horizontal distance covered over the ground surface while walking or running (e.g., 10% slope, in practice 5.71° inclination).

**Fig 2:**
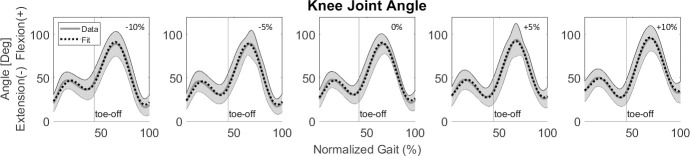
Stage 1: Sagittal knee joint angle as an example of the Fourier series fit for each surface gradient while running at 2.25 m/s using the linear formulation. The dotted line represents the fit, while the gray line represents the average of all participants. The gray shading is the between-participants variability, represented with one standard deviation.

To avoid overfitting, we limited the order of the polynomial to m ≤ 3. Other criteria for determining the size of the polynomial describing the variation in Fourier coefficients as a function of surface gradient were the adjusted R^2^ and relative change in the *β*_*o*_, *δ*_*i*_, *γ*_*i*_ coefficients. The coefficients’ relative change was defined as the total range (maximum–minimum) that a coefficient had at the five surface gradients divided by the difference between the lower-limb joint variables’ maximum and minimum values during a stride cycle. For example, since the largest change in the knee angle was 70° (**Fig 2**) and the knee angle β_*0*_ ranged 48–55°, the relative change for β_*0*_ is 7/70 = 0.1. We used relative change since joint angle, moment, and mechanical power are of different magnitude, and relative change allowed us to apply the same rules for all three.

**Fig 3:**
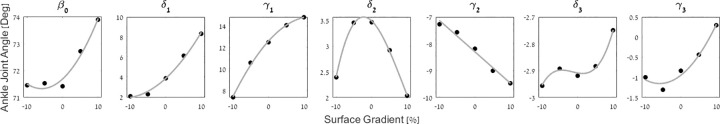
Stage 2: The fitted polynomial for the Fourier coefficients (black dots) as a function of surface gradient (fitted gray line) demonstrated with the ankle angle from running at 2.25 m/s.

The final polynomial size was determined as follows. When the relative change was less than 5%, we performed a linear fit. When the relative change of the coefficients was more than 5% but less than 20%, we compared the polynomial fit that achieved the highest adjusted R^2^ to a polynomial with one order less. If the change in the adjusted R^2^ was less than 5%, we reduced the polynomial order to the lowest order that would result in a less than 5% change in the adjusted R^2^. When the coefficients’ relative change was higher than 20%, we compared the polynomial fit that achieved the highest adjusted R^2^ and a polynomial with one order less. If the change in the adjusted R^2^ was less than 2%, we continued to reduce the polynomial order to the lowest order that would result in a less than 2% change in the adjusted R^2^. **Fig 3** illustrates this set of rules with the ankle angle data. MATLAB (MathWorks, Inc., Natick, MA) codes used to generate the time series of predicted lower-limb joint variables while walking or running at any gradient within the trained range are included and can be downloaded online (S1 Data). In most of the 90 combinations of surface and joint parameter (e.g., hip angle in the frontal plane, while running in -10% slope) the equations were developed from 14 participates. However, in a few parameters due to errors in data collection the data set used to develop the equation was from less than 14 but not less than 9 participants.

The Fourier coefficients in this example are used in **Eq 1** as follows:

$$Ankle\_Angle(x)=\beta_{0}+\delta_{1}\;\sin\left(\omega x\right)+\gamma_{1}\;\cos(\omega x)+\delta_{2}\;\sin\left(2\omega x\right)+\gamma_{2}\;\cos(2\omega x)+\delta_{3}\;\sin\left(3\omega x\right)+\gamma_{3}\;\cos\left(3\omega x\right)$$

Of note: we used a similar approach for each lower-limb joint’s angle, moment, and mechanical power profiles.

### 2.5 Evaluation of the parametric equations

Following the evaluation of the trained data’s prediction equations, we examined their performance using unknown data set aside during the equations’ development. The two types of unknown data examined were from two new participants and four new slopes from 8 BGU participants (1 new and 7 previously utilized data) at each velocity (walking: -12.5%, -5%, +5%, +12.5%; running: -7.5%, -2.5%, +2.5%, +7.5%). We chose to present most the validation figures on the sagittal plane since most of the mechanical work is done on this plane [16].

### 2.6 Effect of participants’ gender on the prediction equations

Additionally, we tested whether a single set of equations accounted for both females and males. The participants were thus separated into two groups based on their gender, with 7 participants in each group. We then developed a set of parametric equations for each group with one thing in common: fixed series sizes, determined previously for the whole group. We then defined a meaningful coefficient (mc) equation (**Eq 5**) to determine the ratio of the difference between each gender’s models to the model’s mean square error:

mc=(∑1n(yfi−ymi)2)n(MSE1+MSE2)2 (5)

where *y*_*f*_ and *y*_*m*_ are the models for lower-limb joint variables in each anatomical plane for the female and male group, respectively; *i* is the index for the surface gradient; *n* is the total number of surface gradients (*n* = 5); and *MSE*_1_ and *MSE*_2_ are the models’ mean square errors. If the mc is 1 or lower, the difference between the models is similar to or smaller than the error in the model’s predication (for each groups), meaning a single model sufficiently explains the data for both groups. Meanwhile, large mc values indicate that the difference between the models is larger than the errors between each model and its experimental data, meaning two separate models are required.

### 2.7 Enabling an extension of our predication equations to estimate joint work as a function of surface gradient

Our prediction equations can be used to study gait even for lower-limb joint variables that we did not fit. Here, we show how to calculate the joint work during a stride cycle from 0 to 100 (analytically or numerically, e.g., Euler):

$$W_{N}=\int_{0}^{100}p_{N}(stride\;cycle\;\%,surface\;gradient)\;dt_{N}\approx \sum_{1}^{100}p_{N}(i)\frac{1}{100}$$ (6)

where *W*_*N*_ is the normalized work, *p*_*N*_ is the normalized power, and *i* is the percentage in the normalized gait cycle (t_N_). Note that *W*_*N*_ can be used to calculated only negative work or only positive. Since our equations predict the mechanical power of a stride cycle normalized to a participant’s height and weight, in order to compute a specific participant’s actual mechanical work (e.g., for the purpose of designing an assistive device), the normalized work needs to be multiplied by stride time, participant height, and participant mass:

$${W=W}_{N}\times ST\times M\times H$$ (7)

where *ST* is stride time, M is participant mass, and H is participant height. Stride time can be calculated using **Eqs 8** or **10**, which we present in the Results section 3.5. To obtain these equations, we developed regression equations for stride cycle time and stride length as a function of surface gradient, with stride length calculated as SL = V×ST (V = treadmill velocity, ST = stride time). Note that *W*_*N*_ is calculated using integration of the power equation; therefore, the adjusted R^*2*^ for the for the power equation of a given joint and plane provides a good estimation of the accuracy of W_N_. Two equations are used when calculating W (**Eq 7**), and we know the fit (R^2^) for each. Thus, a good approximation of the worst accuracy of the calculation of this work would be obtained by multiplying the R^2^ values for both power and stride time.

## 3. Results

### 3.1 Stage 1: Fourier fits for each lower-limb joint variable at each surface gradient

After obtaining the first order approximation for the Fourier series, we applied the set of rules described at the end of section 2.4.1 to determine the final form (size) of the Fourier series for each lower-limb joint variable as a function of stride cycle percentage for a given single surface gradient. The average adjusted R^*2*^ for each joint parameter ranged from 0.98 to 1 (0.999) in the sagittal plane, from 0.92 to 1 in the frontal plane, and from 0.72 to 1 in the transverse plane. (**Tables 1 and 2**). Visually (e.g., knee angle in **Fig 2**), we observed that the fit followed the data well for all lower-limb joint variables.

**Table 1:** Evaluation of the prediction equations for each lower-limb joint variable for each given surface gradient as a function of stride-cycle percentage (Stage 1) in all anatomical planes when running at 2.25 m/s. The adjusted R^2^ and RMSE values are presented as averages across surface gradients and standard deviation (in brackets).

*Stage 1*: *Initial fit*, *f(stride cycle %)*, *running at 2*.*25 m/s*									
	Sagittal plane (x-axis)			Frontal plane (y-axis)			Transverse plane (z-axis)		
Parameter	Series order	$R_{Adj}^{2}$	RMSE	Series order	$R_{Adj}^{2}$	RMSE	Series order	$R_{Adj}^{2}$	RMSE
Ankle Angle [^o^]	3	0.99 (0.01)	0.72 (0.28)	5	0.99 (0.00)	0.33 (0.10)	3.5*	0.99 (0.00)	0.36 (0.06)
Knee Angle [^o^]	2	0.99 (0.00)	1.80 (0.34)	4.5*	0.99(0.01)	0.12 (0.04)	5	1 (0.00)	0.23 (0.09)
Hip Angle [^o^]	3	1 (0.00)	0.37 (0.07)	3	0.99 (0.00)	0.31 (0.08)	5	0.96 (0.02)	0.23 (0.07)
Ankle Moment [Nm/)kg*m)]	3	0.99 (0.00)	0.04 (0.01)	4	1 (0.00)	0.01 (0.00)	3	0.72 (0.3)	0.01 (0.00)
Knee Moment [Nm/)kg*m)]	4	0.99 (0.00)	0.03 (0.01)	3.5*	0.92 (0.12)	0.01 (0.00)	5	0.98 (0.01)	0.01 (0.00)
Hip Moment [Nm/)kg*m)]	4.5*	0.99 (0.00)	0.02 (0.00)	5	1 (0.00)	0.02 (0.01)	4	0.96 (0.04)	0.01 (0.00)
Ankle Power [W/)kg*m)]	5	1 (0.00)	0.08 (0.01)	11.5*	0.99 (0.01)	0.01 (0.00)	12	0.83 (0.2)	0.01 (0.00)
Knee Power [W/)kg*m)]	7	0.99 (0.01)	0.11 (0.05)	13	0.99 (0.01)	0.01 (0.00)	11.5*	0.96 (0.03)	0.01 (0.00)
Hip Power [W/)kg*m)]	8	0.99 (0.00)	0.03 (0.01)	10	0.98 (0.03)	0.02 (0.01)	12.5*	0.97 (0.01)	0.01 (0.00)

*Note: Half values indicate that one of the coefficients (δ_i_, γ_i_) was not statistically significant. Adjusted R^2^ and RMSE mean (standard deviation) values are presented.

**Table 2:** Evaluation of the prediction equations for each lower-limb joint variable for each given surface gradient as a function of stride-cycle percentage (Stage 1) in all anatomical planes when walking at 1.25 m/s. The adjusted R^2^ and RMSE values are presented as averages across surface gradients (standard deviation (in brackets).

*Stage 1*: *Initial fit*, *f(stride cycle %)*, *walking at 1*.*25 m/s*									
	Sagittal plane (x-axis)			Frontal plane (y-axis)			Transverse plane (z-axis)		
Parameter	Series order	$R_{Adj}^{2}$	RMSE	Series order	$R_{Adj}^{2}$	RMSE	Series order	$R_{Adj}^{2}$	RMSE
Ankle Angle [^o^]	5	0.99 (0.01)	0.66 (0.15)	5	0.99 (0.01)	0.35 (0.14)	4	0.99 (0.00)	0.29 (0.04)
Knee Angle [^o^]	3	1 (0.00)	1.06 (0.28)	5	0.98 (0.01)	0.17 (0.04)	5	0.98 (0.01)	0.27 (0.03)
Hip Angle [^o^]	3	1 (0.00)	0.32 (0.05)	3	0.99 (0.00)	0.25 (0.11)	5	0.98 (0.01)	0.24 (0.07)
Ankle Moment [Nm/)kg*m)]	4	0.99 (0.00)	0.03 (0.00)	4	0.98 (0.01)	0.01 (0.00)	5	0.99 (0.00)	0.00 (0.00)
Knee Moment [Nm/)kg*m)]	5	1 (0.00)	0.01 (0.00)	4.5*	0.97 (0.01)	0.01 (0.00)	5	0.97 (0.00)	0.01 (0.00)
Hip Moment [Nm/)kg*m)]	3.5*	0.98 (0.01)	0.03 (0.0)	5	0.99 (0.00)	0.02 (0.01)	4	0.97 (0.01)	0.01 (0.00)
Ankle Power [W/)kg*m)]	7	0.98 (0.01)	0.05 (0.01)	12.5*	0.99 (0.01)	0.00 (0.00)	14.5*	0.97 (0.01)	0.00 (0.00)
Knee Power [W/)kg*m)]	8	0.99 (0.00)	0.03 (0.02)	15	0.99 (0.02)	0.00 (0.00)	12	0.98 (0.01)	0.00 (0.00)
Hip Power [W/)kg*m)]	5.5*	0.99 (0.01)	0.03 (0.00)	11	0.99 (0.01)	0.01 (0. 00)	14	0.98 (0.01)	0.00 (0.00)

*Note: Half values indicate that one of the coefficients (δ_i_, γ_i_) was not statistically significant. Adjusted R^2^ and RMSE mean (standard deviation) values are presented.

### 3.2 Stage 2: Evaluation of equation fits for walking and running at any surface gradient (i.e., final fit)

**Fig 4:**
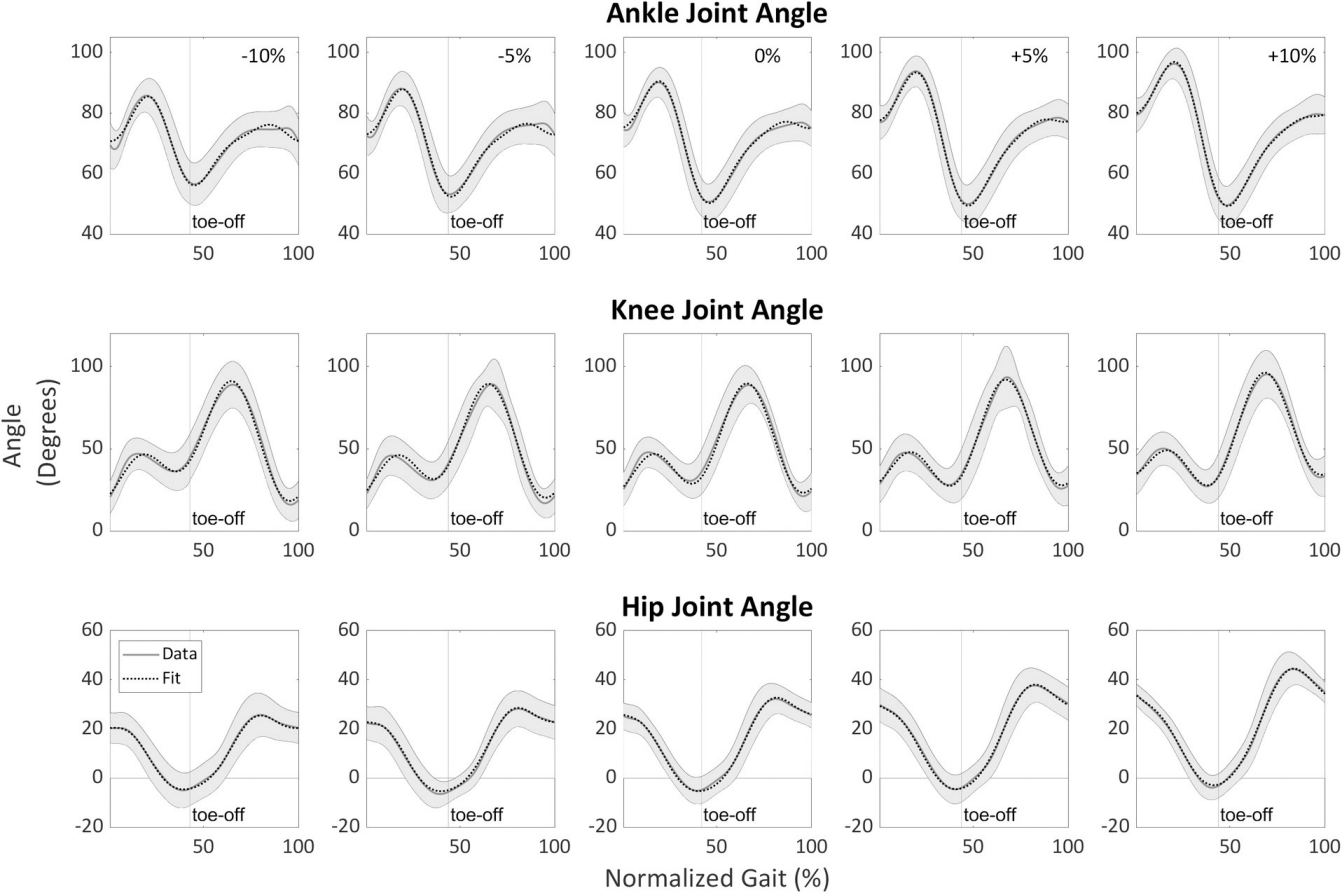
Sagittal plane lower-limb joint angles’ final fits (Stage 2) for running at 2.25 m/s as a function of surface gradient (-10%, -5%, 0%, +5%, +10%) and stride cycle percentage (0% is heel strike) compared with experimental data. The dotted line represents fit, while the gray line represents average data across all participants. The gray shading is the between-participants variability represented with one standard deviation.

**Fig 5:**
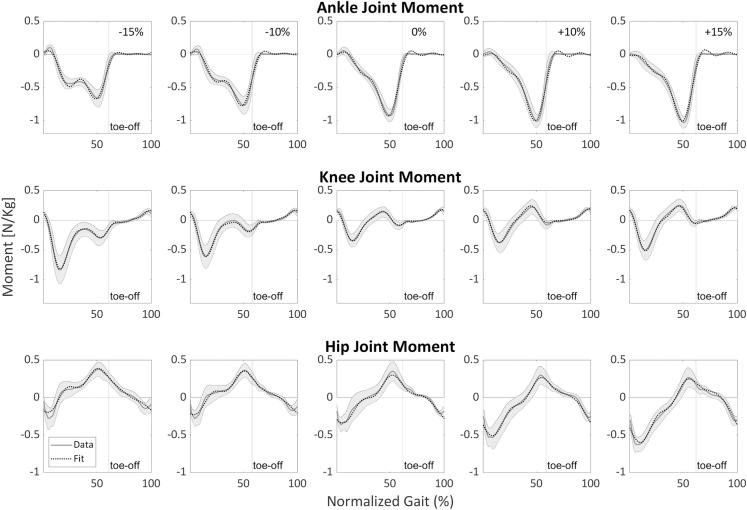
Sagittal plane lower-limb joint moments’ final fits for walking at 1.25 m/s as a function of surface gradient (-15%, -10%, 0%, +10%, +15%) and stride cycle percentage (0% is heel strike) compared with experimental data. The dotted line represents fit, and the gray line represents average data across all participants. The gray shading is the between-participants variability represented with one standard deviation. The moments are normalized by the participants’ height and mass.

**Fig 6:**
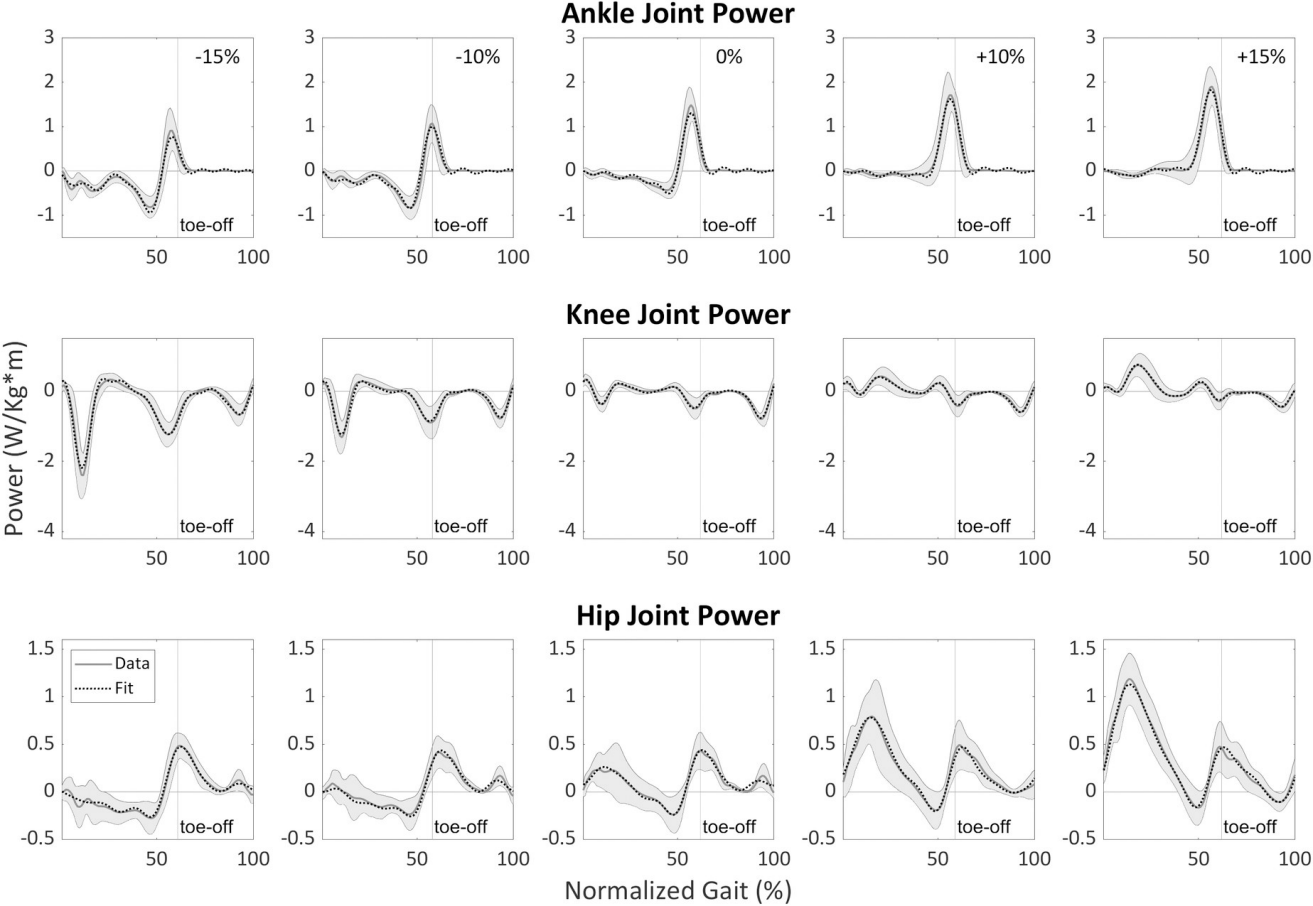
Sagittal plane lower-limb joint mechanical powers’ final fits for walking at 1.25 m/s as a function of surface gradient (-15%, -10%, 0%, +10%, +15%) and stride cycle percentage (0% is heel strike) compared with experimental data. The dotted line represents fit, and the gray line represents average data across all participants. The gray shading is the between- participants variability represented with one standard deviation. The powers are normalized by the participants’ height and mass.

**Fig 7:**
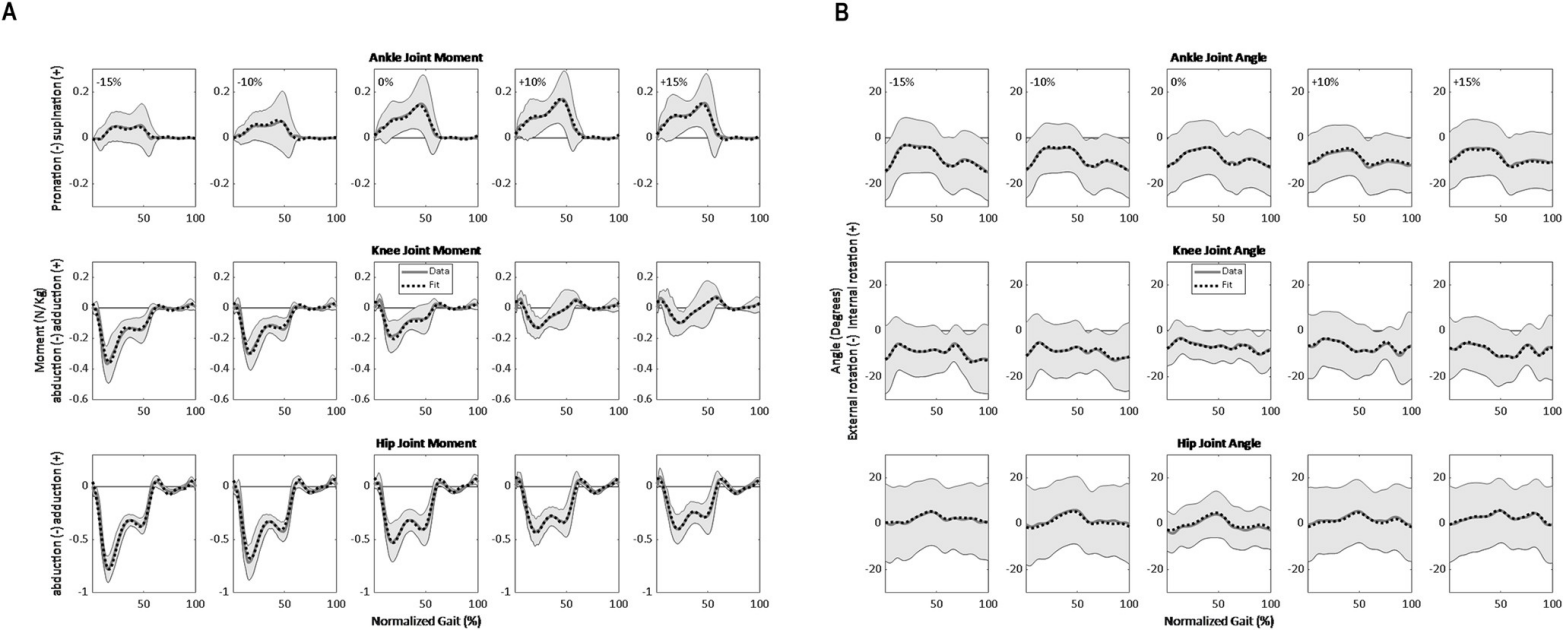
Examples of fit results in the frontal and transverse planes for walking at 1.25 m/s. A: Ankle, knee, and hip moment in the frontal plane (y-axis). B: Ankle, knee, and hip angle in the transverse plane (z-axis). The gray area represents ± 1 standard deviation.

Evaluating the equation fits to predict the lower-limb joint variables for any given surface gradient as a function of stride cycle percentage revealed average adjusted R^2^ values ranging from 0.98 to 1 (0.998) in the sagittal plane, from 0.66 to 0.99 in the frontal plane, and from 0.72 to 0.98 in the transverse plane. As expected, the RMSE values for the final fit (**Tables 3** and **4**) were slightly worse than the fit of each surface gradient (i.e., Stage 1). Visually, the fits followed the data quite well (see **Figs 4–6** for examples in the sagittal plane and **Fig** 7 for examples in the frontal and transverse planes). **S1 File** presents an example of how to assemble these equations. Low values of adjusted R^*2*^ are due to failure of the algorithm to fit the data, see **S2 File** for further explanation and examples.

**Table 3:** Evaluation of the final fit (Stage 2) equations for each lower-limb joint variable, averaged on surface gradients, for running at 2.25 m/s.

Final fit, f(slope, stride cycle %), running at 2.25 m/s									
	Sagittal plane (x-axis)			Frontal plane (y-axis)			Transverse plane (z-axis)		
Parameter	Series order	$R_{Adj}^{2}$	RMSE	Series order	$R_{Adj}^{2}$	RMSE	Series order	$R_{Adj}^{2}$	RMSE
Ankle Angle [^o^]	3	1	0.78	5	0.99	0.37	3.5*	0.90	1
Knee Angle [^o^]	2	0.99	1.9	4.5*	0.66	0.68	5	0.97	0.62
Hip Angle [^o^]	3	1	0.53	3	0.99	0.42	5	0.77	0.59
Ankle Moment [Nm/)kg*m)]	3	0.99	0.04	4	0.98	0.01	3.5*	0.72	0.01
Knee Moment [Nm/)kg*m)]	4	0.99	0.03	3.5*	0.93	0.02	5	0.98	0.01
Hip Moment [Nm/)kg*m)]	4.5*	0.99	0.03	5	0.99	0.03	4	0.96	0.01
Ankle Power [W/)kg*m)]	5	0.99	0.1	11.5*	0.99	0.01	12	0.80	0.01
Knee Power [W/)kg*m)]	7	0.98	0.12	13	0.94	0.01	11.5*	0.85	0.01
Hip Power [W/)kg*m)]	8	0.98	0.04	10	0.91	0.08	12.5*	0.92	0.01

*Note: Half values indicate that one of the coefficients (δ_i_, γ_i_) was not statistically significant.

**Table 4:** Evaluation of the final fit (Stage 2) equations for each lower-limb joint variable, averaged on surface gradients, for walking at 1.25 m/s.

Final fit, f(slope, stride cycle %), walking at 1.25 m/s									
	Sagittal plane (x-axis)			Frontal plane (y-axis)			Transverse plane (z-axis)		
Parameter	Series order	$R_{Adj}^{2}$	RMSE	Series order	$R_{Adj}^{2}$	RMSE	Series order	$R_{Adj}^{2}$	RMSE
Ankle Angle [^o^]	5	0.99	0.77	5	0.97	0.57	4	0.96	0.55
Knee Angle [^o^]	3	1	1.15	5	0.72	0.61	5	0.76	0.94
Hip Angle [^o^]	3	1	0.74	3	0.99	0.43	5	0.93	0.51
Ankle Moment [Nm/)kg*m)]	4	0.99	0.03	4	0.97	0.01	5	0.93	0.00
Knee Moment [Nm/)kg*m)]	5	0.99	0.02	4.5*	0.97	0.01	5	0.98	0.01
Hip Moment [Nm/)kg*m)]	3.5*	0.98	0.03	5	0.99	0.02	4	0.96	0.01
Ankle Power [W/)kg*m)]	7	0.98	0.05	12.5*	0.91	0.01	14.5*	0.92	0.00
Knee Power [W/)kg*m)]	8	0.98	0.04	15	0.82	0.01	12	0.88	0.01
Hip Power [W/)kg*m)]	5.5*	0.98	0.03	11	0.94	0.02	14	0.91	0.01

*Note: Half values indicate that one of the coefficients (δ_i_. γ_i_) was not statistically significant.

### 3.3 Evaluation of the parametric equations

**Fig 8:**
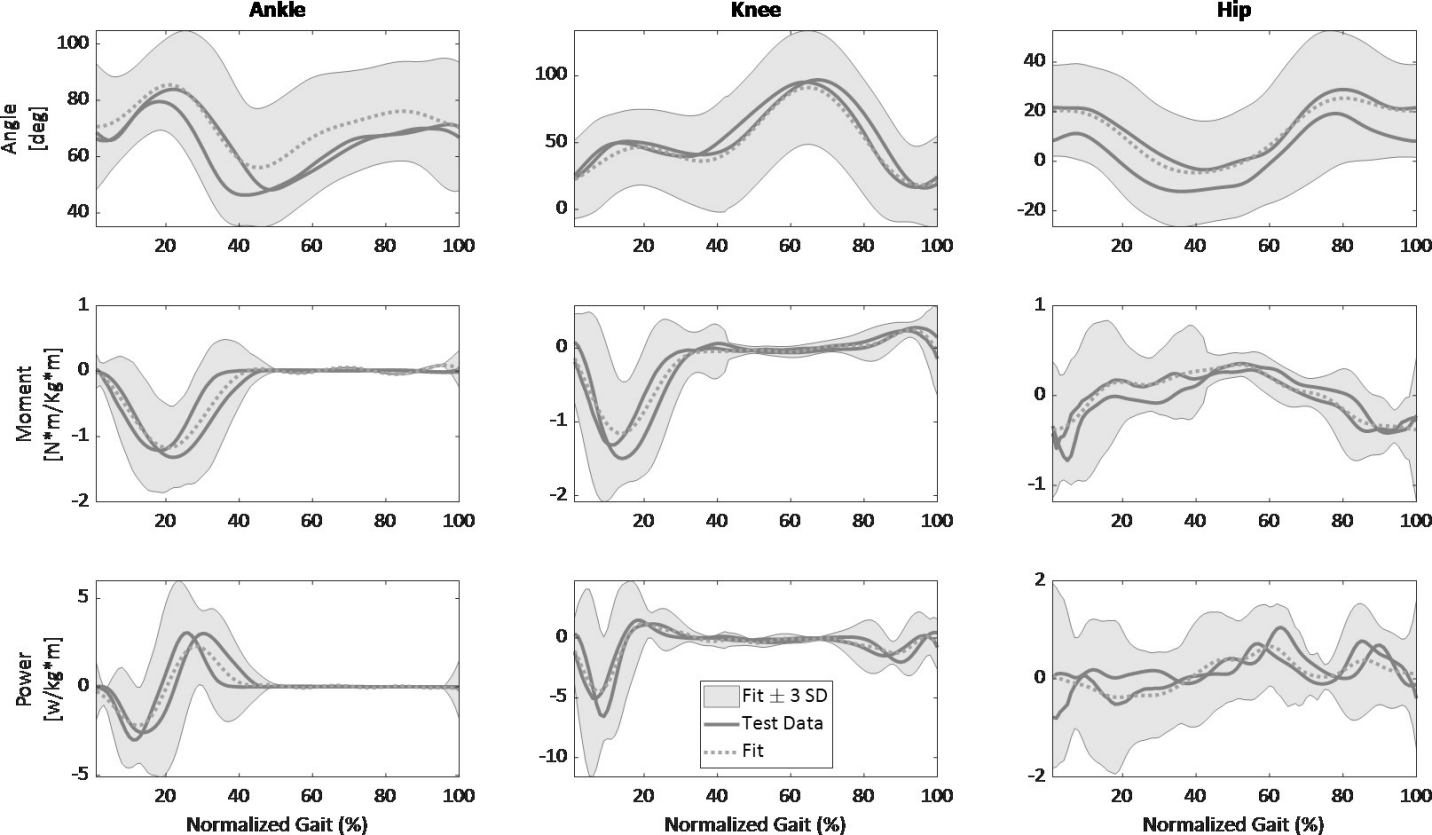
Comparison between the final fits and data on the sagittal plane from two participants not included in the model’s training, featuring lower-limb joint variables for running at a -10% surface gradient as a function of stride cycle percentage (0% is heel strike) compared with experimental data. The dotted line represents fit, and the gray line represents the two test participants’ average cycle data. The gray shading is the between- participants variability represented with three standard deviations.

**Fig 8** demonstrates an example of the relations between the prediction equations and new data. In this example, we plotted the model (fit) within a range of three standard deviations (this range should include 99% of the population variation) calculated from the training data. We expected the test data to fall within that range, as can be seen in the example. As for the slopes not used for the equations’ development, our evaluation revealed that when examining all slopes on the three planes, the mean adjusted R^2^ is 0.94 (range: 0.86–0.98) and mean RMSE 0.84 (range: 0.03–2.85) for walking, and the mean adjusted R^2^ is 0.89 (range: 0.74–0.98) and mean RMSE 1.55 (range: 0.07–7.7) for running.

### 3.4 Effect of participants’ gender on the prediction equations

**Fig 9:**
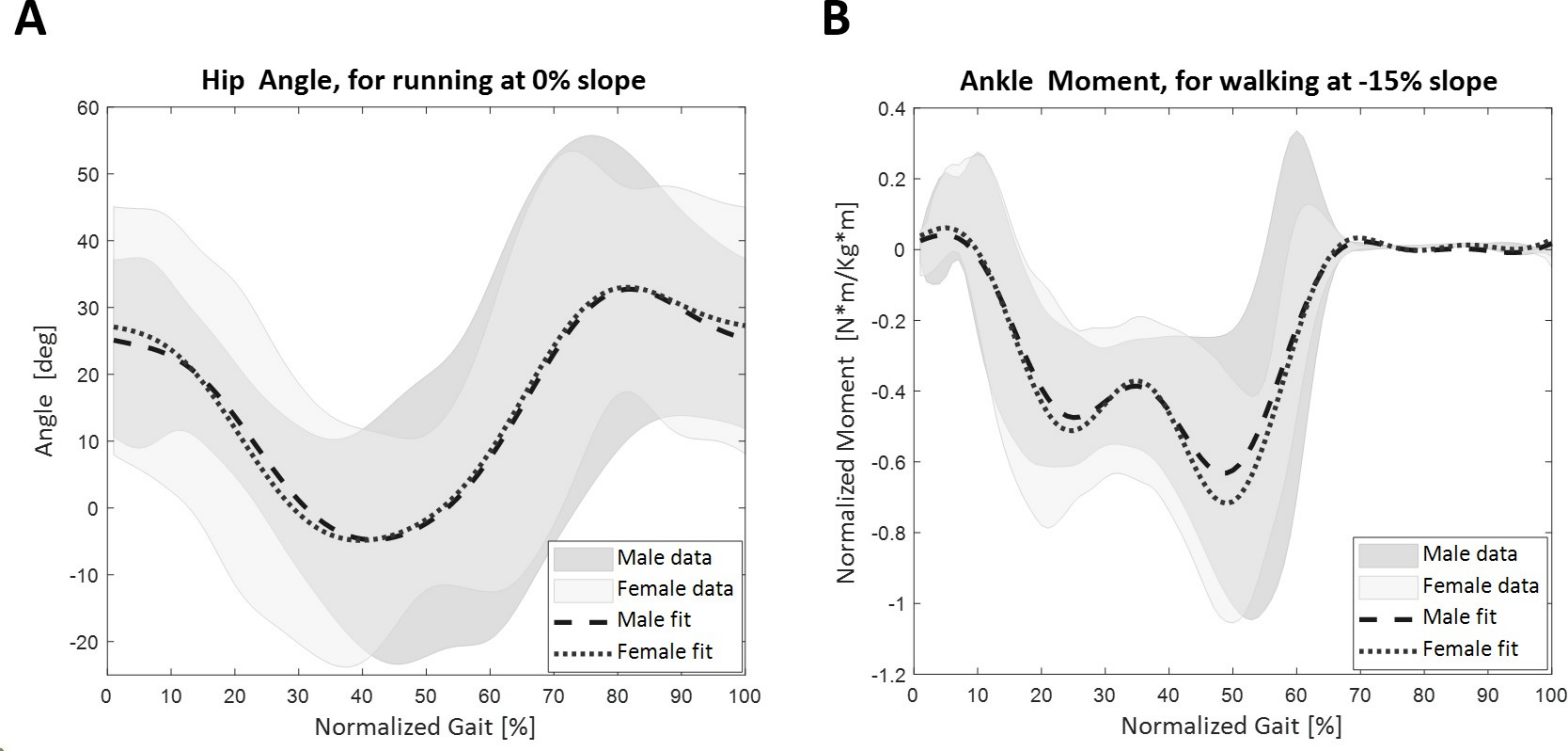
Examples and data distributions of the two models for each gender (male and female), with the dark and light gray shading represent averaged signal ± 3 standard deviations for male and female participants, respectively. A: Data and fit of the hip angle in the sagittal plane while running at 2.25m/s at a 0% slope. B: Data and fit of the ankle moment in the sagittal plane while walking at 1.25m/s at a -15% slope.

When testing for the effect of gender, we found that the meaningful coefficients (**Eq 5**) were lower than 1 for all but one of the lower-limb joint variables (hip angle in the frontal plane). This indicated that the differences between the two genders’ models were smaller than the errors between the experimental data and models themselves. A visual inspection also showed the models were qualitatively similar (**Fig 9**). The only exception was that the models for the hip angle in the frontal plane had an mc greater than 1 for walking (mc = 1.385) and very close to 1 for running (mc = 0.962).

### 3.5 Stride cycle time and length as a function of surface gradient

One use of the stride-cycle time is to calculate joint work (**Eq 7**) since the lower-limb joint variable predicted using the predication equation are time-normalized. We fitted polynomial regression equations for both stride time and length for running (**Eqs 8** and **9**, R^2^ = 0.95) and walking (**Eqs 10** and **11**, R^2^ = 0.99):

StrideTime=0.7361−0.0006∙SG (8)

StrideLength=1.6561−0.0014∙SG (9)

$$Stride\;Time=0.0039+0.0006∙SG-1{∙10}^{-4}∙{SG}^{2}$$ (10)

$$Stride\;Length=1.3574+0.0049∙SG-1{∙10}^{-4}∙{SG}^{2}$$ (11)

where *SG* is the Surface Gradient (%). Examination of the average stride time revealed that running cycle time doesn’t change as a function of the surface gradient, while walking cycle time increase as the surface gradient is increased (**Tables 5** and **6**).

**Table 5:** Running (2.25 m/s) cycle time (s) over a range of surface gradients.

	Surface Gradient (%)				
	-10	-5	0	5	10
Average	0.74	0.74	0.74	0.73	0.73
Between- participants standard deviation	0.03	0.04	0.04	0.04	0.03
Within- participants average standard deviation	0.02	0.01	0.01	0.01	0.01

**Table 6:** Walking (1.25 m/s) cycle time (s) over a range of surface gradients.

	Surface Gradient (%)				
	-15	-10	0	10	15
Average	1.00	1.04	1.08	1.12	1.11
Between- participants standard deviation	0.05	0.05	0.05	0.07	0.06
Within- participants average standard deviation	0.03	0.02	0.02	0.03	0.03

### 3.6 Estimation of joint work as a function of surface gradient

**Fig 10:**
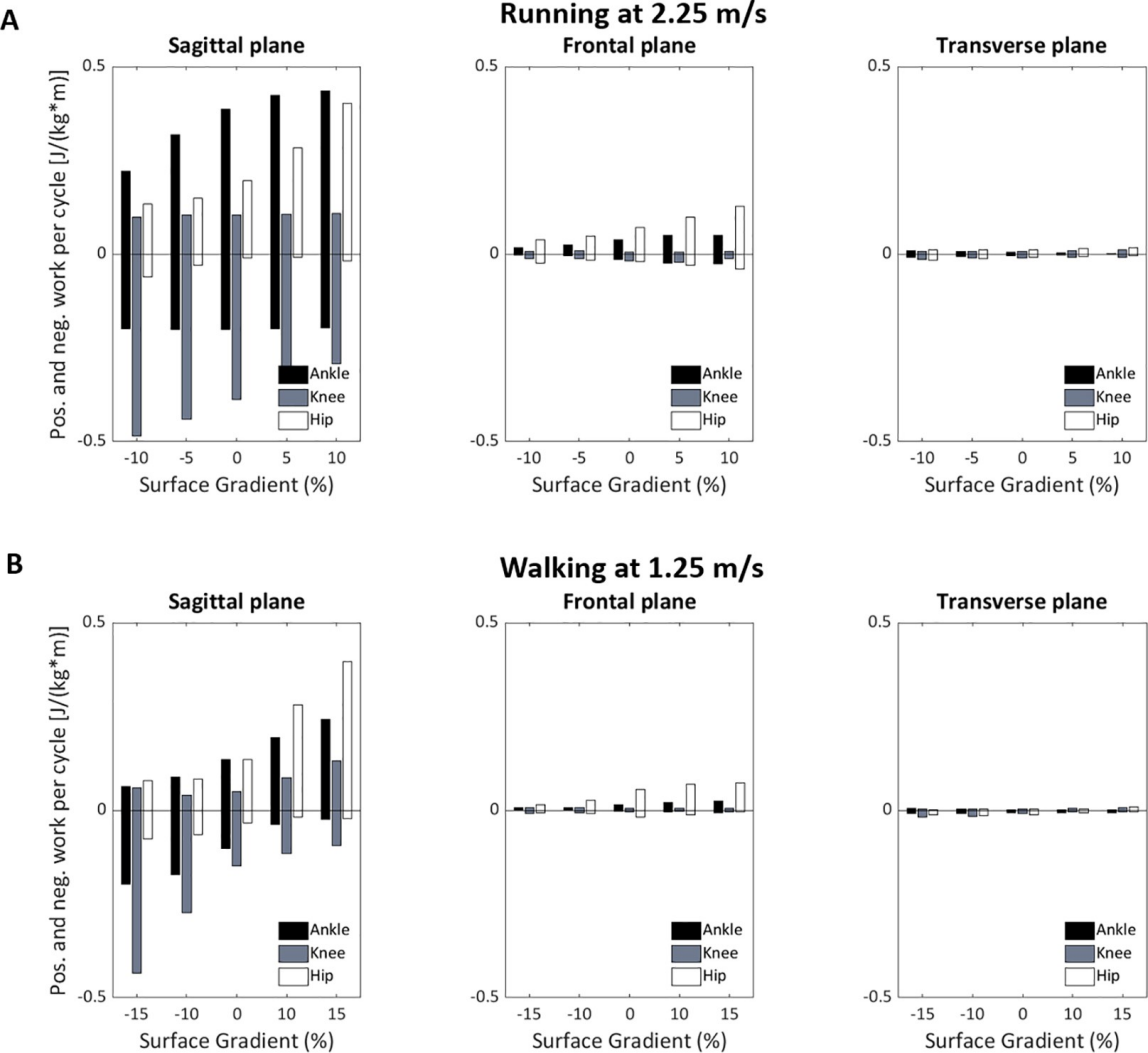
Positive and negative work per stride cycle as a function of the surface gradient in three anatomical planes (top panel: Running; bottom panel: Walking). The bar above zero represents positive work performed in the joint during a stride cycle; the bar below zero represents negative joint work.

This section presents the potential use of the prediction equations for estimating joint work. Normalized joint work was calculated using **Eq 6** and then multiplied by average stride time estimated in section 3.4, producing the average work per stride cycle. This joint work estimation was calculated for each joint (in three anatomical planes for both walking and running) as a function of the surface gradient (**Fig 10**). The results reveal that most of the walking and running work is performed in the sagittal plane and that the amount of positive work increases as slopes change from negative to positive. Also, when walking on negative slopes, most of the negative work in the sagittal plane is performed by the knees; on positive slopes, most of the positive work is performed by the hips. When running, the knee provides a great amount of negative work, regardless of the slope, while the ankle’s negative joint work is relatively constant. Moving from negative to positive slopes increases the positive work. Hips provided relatively small negative joint work and larger positive work, which both increase as the runners move from negative to positive slopes. In the frontal plane, the hips show a change in the amount of negative and positive joint work as the slope angle increases.

## 4. Discussion

In this study, we develop prediction equations for average lower-limb joint kinematics and kinetics time series data over a stride cycle during human walking (1.25 m/s) and running at a slow speed (2.25 m/s) on different surface gradients. These equations could be useful in many applications, such as a reference for an exoskeleton, a prosthesis, or a walking robot controller as well as for device optimization. They may also be useful as an educational tool, enabling students to produce joint parameters of many gait states and analyze them, tasks that would be much harder to perform using raw data.

The experimental lower-limb joint angle, moment, and mechanical power data for hip, knee, and ankle show similar patterns to previous sagittal plane data [16,22,31]. We also developed a separate set of equations for each anatomical plane. The final fit Fourier equations reached high adjusted R^2^ values for most parameters. The best adjusted R^2^ values were in the sagittal plane and slightly less in the frontal and transverse planes, indicating a high goodness-of-fit. In several parameters in the frontal and transverse planes (e.g., knee angle; see **Tables 3** and **4**),

Most of the sources of poor fit are due to the first coefficients of the polynomial regression, which are not well fitted (**S2 File, S2A Fig**). Thus, while the final fit follows the averaged signal, there is an offset at each slope (**S2B Fig**). This results in low adjusted R^2^ values, even though the initial fit for each slope was very good individually.

With regard to the participants’ gender effect on the prediction equations, we found that only hip angle in the frontal plane was affected. This is in line with previous studies, which found gender-related differences in hip adduction during walking and running [32–34]. These differences occur during the termination of the swing phase and initiation of the stance phase for both walking and running. Given that women show larger hip adduction compared to men, we developed additional models for the walking and running hip angles of men and women separately (see S1 Data). However, since the overall similar motion patterns and variability between participants (see **S3 Fig**), we also provided a prediction equation for men and women collectively. It should be mentioned that the group sizes were relatively small (7 participants of each gender), and a larger sample could provide more parameters in which men and women are statistically different.

Despite the general success of our approach, in terms of high R^2^, the use of data for only one walking and one running speed may have limited its utility. Hence, future studies should extend our approach to different speeds as an input parameter. For instance, **Eqs 2–4** can be modified as follows:

$$\beta_{0}=a_{0}+\sum_{j=1}^{m}a_{j}\times {SG}^{j}+b_{j}\times S^{j}+c_{j}{{\times SG}^{j}\times S}^{j}$$ (12)

$$\delta_{i}=d_{i0}+\sum_{j=1}^{m}d_{ij}\times {SG}^{j}+e_{ij}\times S^{j}+f_{ij}{{\times SG}^{j}\times S}^{j}$$ (13)

$$\gamma_{i}=g_{i0}+\sum_{j=1}^{m}g_{ij}\times {SG}^{j}+h_{ij}\times S^{j}+k_{ij}{{\times SG}^{j}\times S}^{j}$$ (14)

where *SG* is the surface gradient, *S* is running or walking speed, *i* is the index for the coefficients from **Eq 1**, j is the index of the polynomial component, and *m* is the polynomial order.

During the experiment, the type of running (i.e., rearfoot, forefoot or midfoot running) was not controlled. However, post hoc examination of the data revealed that one of the participants was a forefoot runner while the rest were rearfoot runners; moreover, three of the rearfoot runners ran using forefoot or midfoot strikes on the greatest slopes (10%, -10%). The prediction equations were developed using a mixture of these running types and therefore represent the participants average behavior. Future work can focus on the differences between runners and the effect of the foot-strike on the prediction equations.

Notably, this study utilized a single foot segment to calculate ankle kinetics and kinematics. However, it was shown that using a more complex multi-segment model of the foot results in slightly different ankle kinematics and power output, while the torque stays extremely similar in the sagittal plane [35–39]. This, however, does not affect the knee and hip calculations.

As described in the Methods section, this study was conducted in two different facilities (BGU and NCSU) by different staff using different equipment. By using the same marker set (despite collecting the data in two different labs with different types of equipment and different staff), yet the only correction that was applied was for the knee angle offset. This is due to the markers being placed in slightly different locations by the staff at the two labs, which led to a small bias in the averaged knee-joint angle but not the angle profile. This offset was corrected via substruction of the mean and affects only the angle and no other kinematics and kinetics variables. Note that, in theory, the changing marker placement might cause rotation of the joint coordinate system, and this will affect the angles at all three planes.

This study utilized a very common model (CAST [25]) for measuring human motion; however, this is not the only available model. A comparison of five prevalent models reveled that in the sagittal panel, the trajectories of all five models were similar (although in some of them, an offset correction was required). Yet, in the transverse and frontal planes, for several parameters, some of the models produced similar results while others did not [30].

Although the study participants were fairly young (24.56 ± 3.16 years), the literature shows that the kinetics and kinematics in the lower-limb joints were similar until approximately 40 years old [40,41]. Moreover, since the variability between participants is large in comparison to the differences across ages, it is highly likely that older participants display similar walking and running patterns [40,41]. Thus, the prediction equations could be used (with caution) for older, healthy populations.

Our predictive model equations with simple parameter input structures can generate time series data over a surface gradient continuum that may otherwise require exhaustive data collection and analysis. For example, our equations can be used to simulate or generate joint dynamics data for walking and slow human running when a properly equipped gait laboratory is not available. Similarly, our prediction equations can save the time and effort required to directly obtain, through measurement, secondary metrics based on lower-limb joint dynamics, including mechanical work (section 3.6), range of motion (minimum and maximum values of the angle profiles), and joint stiffness (movement as a function of the joint angle). Metrics such as these might prove useful for setting lower-limb orthosis and prosthesis design requirements, as well as those for other wearable devices that assist or augment human locomotion. Finally, our equations may also prove very useful in any application that requires comparison to an average normative data set, such as when assessing changes in function due to musculoskeletal or neurological impairment. However, they may not be useful when the effect of the impairment is only on gait variability Furthermore, this approach and our code (in the S1 Data) can be utilized in biomechanical classes for comparison to inverse dynamics results, and also to enable several analyses without a gait lab (e.g., find which joint provides the most positive work when walking on a 10% slope, explore how the knee-joint work changes on negative to positive slopes when walking, etc.).

Lastly, it is possible that our approach may trigger the creation of an open-source digital library that extends the ideas put forth previously in textbook appendices [15] and online data sources [42,43] to compile data sets for human locomotion researchers to draw upon. For example, models with the framework presented here can be constantly improved with new data tagged by several salient input parameters (e.g., gait mode, speed, surface gradient), then collected into a central digital location from many different laboratories.

## Supporting information

S1 Fig Marker placements based on the CAST marker set [25]. The group of markers named RTH1, RTH2, RTH3, and RTH4, the group named RSK1, RSK2, RSK3, and RSK4, and the markers on the back (RPS, LPS, IP) are rigid clusters of markers placed on acrylic glass plates.
(TIF)

S2 Fig Demonstrates an example in which a participant’s knee-joint angle in the frontal plane displays an averaged adjusted R^2^ of 0.66. A: First five coefficients from the prediction equation of the knee-joint angle in the frontal plane, where the black dots represent the value of each slope, and the gray line is the fit for that coefficient. Note that for *β*_*o*_, the fit has an error in the order of 1 degree. B: Final fit when running at five different slopes. The gray line represents the average between participants, and the dotted line represents the predicted signal (fit); there is a constant offset (due to the difference in *β*_*o*_ and the average signal).
(TIF)

S3 Fig Differences in the separate male and female participant models, hip angle on the frontal plane (y-axis); A: Walking; B: Running. The gray lines represent male participants, and the black lines represents female participants. Each line represents a slope’s average. Note that the main differences between genders appear at the beginning and toward the end of the gait, with the gait cycle measured from heal strike to heal strike.
(TIF)

S1 Table Lower-limb joint angle orientations: Direction and type of movement in three anatomical planes. (DOCX)

S1 File An example of using the final fit equations to predict lower-limb joint variables. (DOCX)

S2 File Prediction equations in three anatomical planes: Examination of difficulties in modeling joint variables (i.e., angles, moment, power). (DOCX)

S3 File Content of supplementary data. (DOCX)

S1 Data (RAR)
