# Supplementary figures and images for: Parametric equations to study and predict lower-limb joint kinematics and kinetics during human walking and slow running on slopes
Source: PLoS One. 2022 Aug 4;17(8):e0269061. doi: 10.1371/journal.pone.0269061 (PMC9352080; doi:10.1371/journal.pone.0269061)

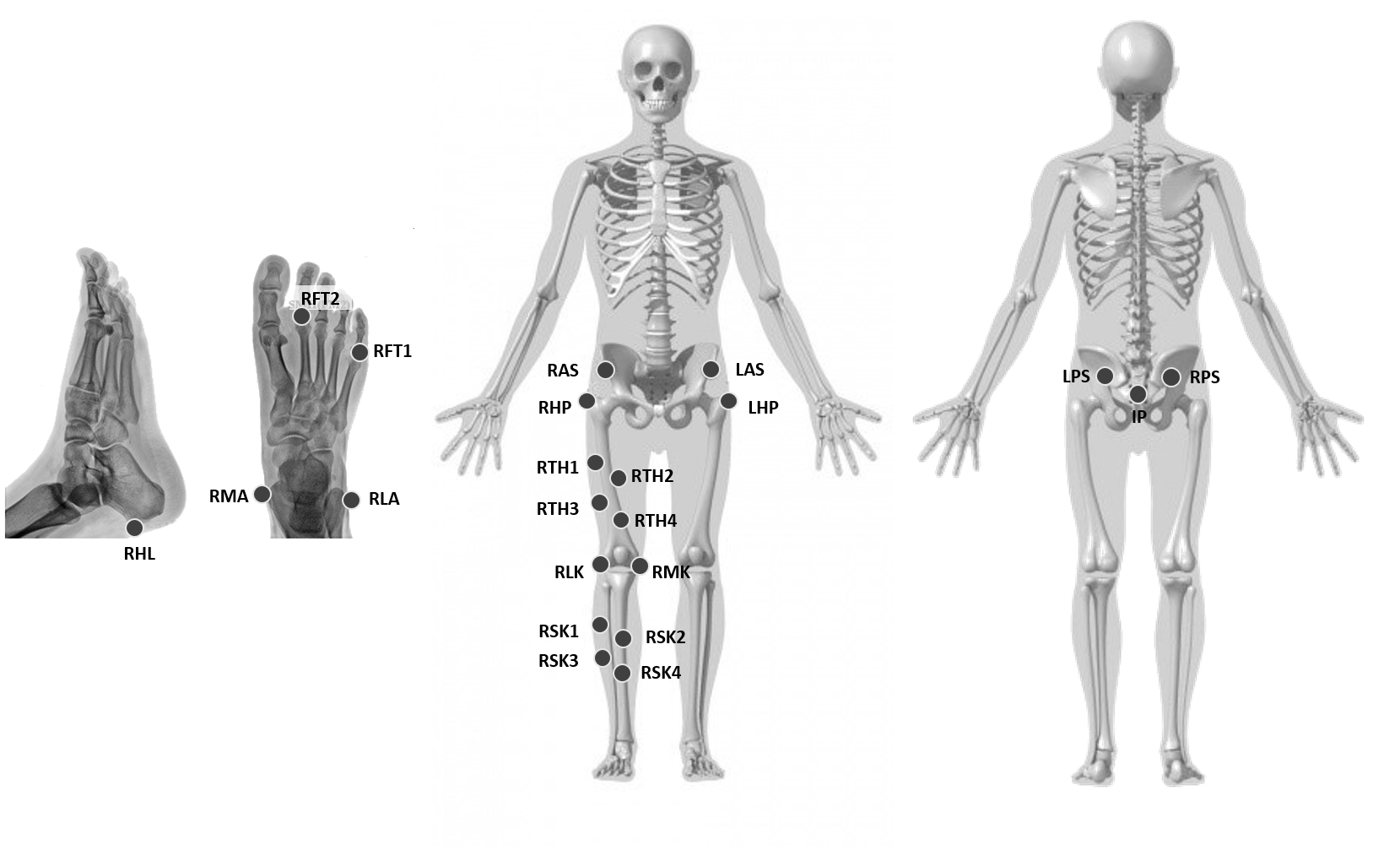

Supplement: S1 Fig — The group of markers named RTH1, RTH2, RTH3, and RTH4, the group named RSK1, RSK2, RSK3, and RSK4, and the markers on the back (RPS, LPS, IP) are rigid clusters of markers placed on acrylic glass plates. (TIF) [file pone.0269061.s001.tif]

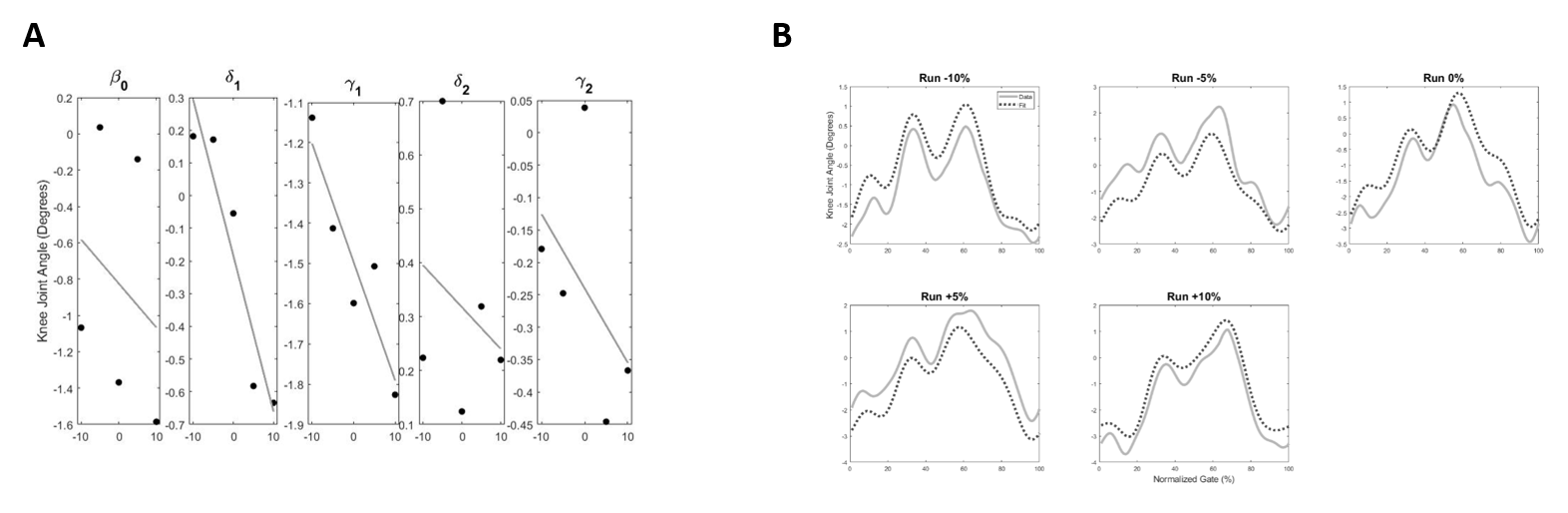

Supplement: S2 Fig — A: First five coefficients from the prediction equation of the knee-joint angle in the frontal plane, where the black dots represent the value of each slope, and the gray line is the fit for that coefficient. Note that for βo, the fit has an error in the order of 1 degree. B: Final fit when running at five different slopes. The gray line represents the average between participants, and the dotted line represents the predicted signal (fit); there is a constant offset (due to the difference in βo and the average signal). (TIF) [file pone.0269061.s002.tif]

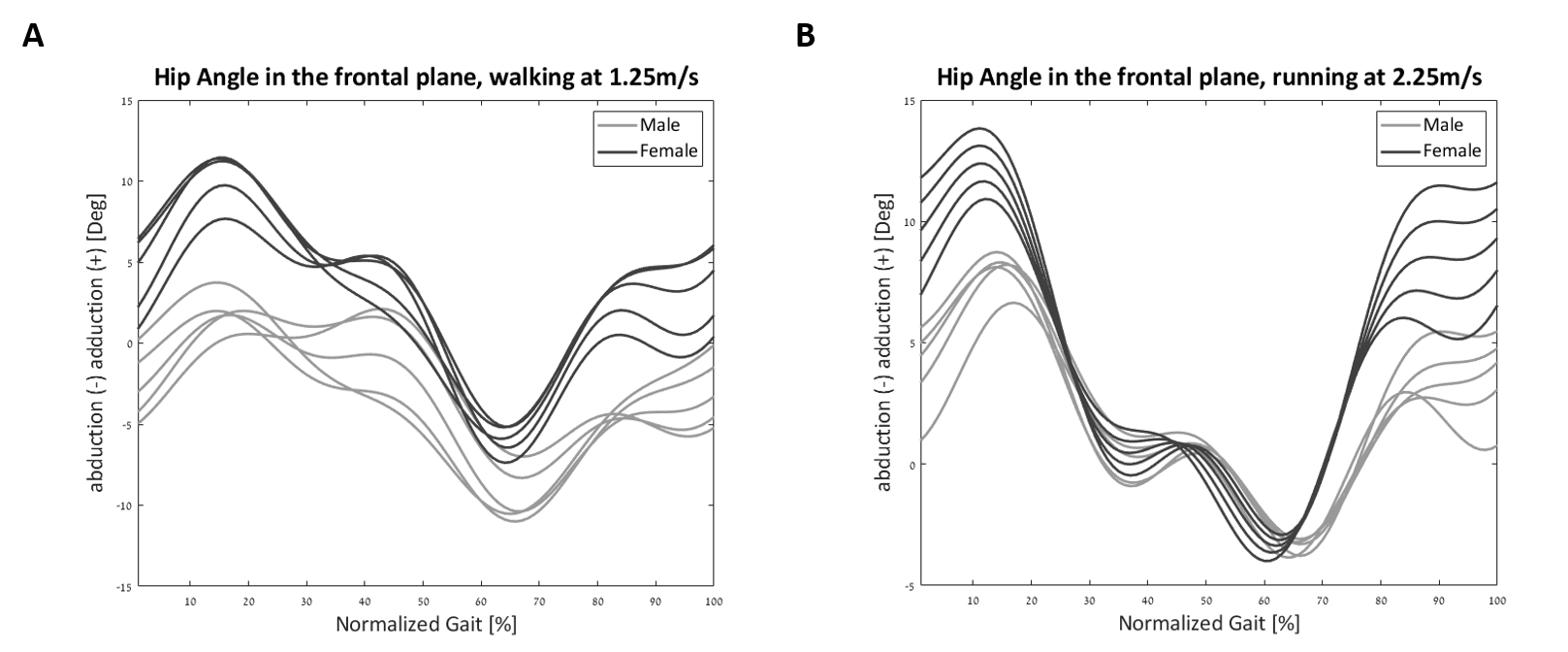

Supplement: S3 Fig — Differences in the separate male and female participant models, hip angle on the frontal plane (y-axis); A: Walking; B: Running. The gray lines represent male participants, and the black lines represents female participants. Each line represents a slope’s average. Note that the main differences between genders appear at the beginning and toward the end of the gait, with the gait cycle measured from heal strike to heal strike. (TIF) [file pone.0269061.s003.tif]
