# Supplementary material for: Parametric equations to study and predict lower-limb joint kinematics and kinetics during human walking and slow running on slopes
Source: PLoS One. 2022 Aug 4;17(8):e0269061. doi: 10.1371/journal.pone.0269061 (PMC9352080; doi:10.1371/journal.pone.0269061)
Supplement: S1 Table — (DOCX) [file pone.0269061.s004.docx]

**S1 Table.** Lower-limb joint angle orientations: Direction and type of movement in three anatomical planes.

| *Movement Orientation* | | | |
| --- | --- | --- | --- |
|  | ***Ankle*** | ***Knee*** | ***Hip*** |
| *Sagittal* | Dorsi-flexion (+) | Flexion (+) | Flexion (+) |
|  | Plantar-flexion (-) | Extension (-) | Extension (-) |
| *Frontal* | Supination (+) | Abduction (-) | Abduction (-) |
|  | Pronation (-) | Adduction (+) | Adduction (+) |
| *Transverse* | External rotation (-) | External rotation (-) | External rotation (-) |
|  | Internal rotation (+) | Internal rotation (+) | Internal rotation (+) |
