# Supplementary material for: Parametric equations to study and predict lower-limb joint kinematics and kinetics during human walking and slow running on slopes
Source: PLoS One. 2022 Aug 4;17(8):e0269061. doi: 10.1371/journal.pone.0269061 (PMC9352080; doi:10.1371/journal.pone.0269061)
Supplement: S1 File — (DOCX) [file pone.0269061.s005.docx]

*An example of using the final fit equations to predict lower-limb joint variables*

We provide here an example of using the final fit equations to predict ankle angle while running (2.25 m/s) as a function of surface gradient and stride cycle percentage. We do recommend applying regression-based models to predict values in the surface gradients’ ranges these models were based on (for running, from -10% to 10%, for walking, from -15% to 15%).

(1) $\beta_{0}+\sum_{i=1}^{n} \left[ \delta_{i}\times\sin\left( i\omega x \right)+\gamma_{i}\times\cos\left( i\omega x \right) \right]$

(2) ${\beta_{0}=\sum_{j=0}^{m} \sigma_{j}\times SG}^{j}$

(3) $\delta_{i}=\sum_{j=0}^{m} \rho_{ij}\times{SG}^{j}$

(4) $\gamma_{i}=\sum_{j=0}^{m} \mu_{ij}\times{SG}^{j}$

where $=\frac{2\pi}{100}$ , *x* is stride cycle (%), and *SG* is surface gradient (%).


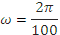


Below is a demonstration of how to calculate ankle joint angle where, for example, we assume that *SG* is 10% and *x* is 50%. The Fourier parameters (β ,δ, γ) for ankle angle to be used in **Eqs. 2–4**) are listed in **S2** **Table**. (Tables for calculating other gait parameters (e.g., knee angle, hip moment, etc.) can be found in the Electronic Supplementary Material.)

$$\beta_{0}=71.6897+0.1219\cdot SG+0.0104\cdot{SG}^{2}=73.9444$$

$$\delta_{1}=3.8647+0.3267\cdot SG+0.0134\cdot{SG}^{2}=8.4758$$

$$\gamma_{1}=12.5843+0.3688\cdot SG+(-0.0148)\cdot{SG}^{2}=14.7929$$

$$\delta_{2}=3.4876+\left( -0.0653 \right)\cdot SG+\left( -0.0127 \right)\cdot{SG}^{2} +0.00047\cdot{SG}^{3}=2.0359$$

$$\gamma_{2}=-8.2986+\left( -0.1173 \right)\cdot SG=-9.4711$$

$$\delta_{3}=-2.9086+\left( -0.0024 \right)\cdot SG+0.00057\cdot{SG}^{2} +0.00013\cdot{SG}^{3}=-2.7487$$

$$\gamma_{3}=-0.9474+0.069\cdot SG+0.0058\cdot{SG}^{2}=0.3213$$

The final fit Fourier equation yields for ankle angle ($\omega=\frac{2\pi}{100}$) at x = 50% are thus:

$$\theta_{Ankle}=73.9444+8.4758\sin\left( \omega x \right)+14.7929\cos\left( \omega x \right)+2.0359\sin\left( 2\omega x \right)+(-9.4711)\cos\left( 2\omega x \right)+(-2.7487)\sin\left( 3\omega x \right)+0.3213\cos\left( 3\omega x \right)=49.359$$

**S2 Table.** Ankle angle (°): Coefficients for Fourier prediction equations as a function of surface gradient (%) in the sagittal plane while running at 2.25 m/s.

| Surface Gradient (SG) | | | |  |
| --- | --- | --- | --- | --- |
| *j = 3* | *j = 2* | *j =1* | *j = 0* | Coefficient |
| 0 | 0.0104 | 0.1219 | 71.6897 | $\sigma_{0}$ |
| 0 | 0.0134 | 0.3267 | 3.8647 | $\rho_{1}$ |
| 0 | -0.0148 | 0.3688 | 12.5843 | $\mu_{1}$ |
| 0.00047 | -0.0127 | -0.0653 | 3.4876 | $\rho_{2}$ |
| 0 | 0 | -0.1173 | -8.2986 | $\mu_{2}$ |
| 0.00013 | 0.00057 | -0.0024 | -2.9086 | $\rho_{3}$ |
| 0 | 0.00578 | 0.0690 | -0.9474 | $\mu_{3}$ |
