# Supplementary material for: Parametric equations to study and predict lower-limb joint kinematics and kinetics during human walking and slow running on slopes
Source: PLoS One. 2022 Aug 4;17(8):e0269061. doi: 10.1371/journal.pone.0269061 (PMC9352080; doi:10.1371/journal.pone.0269061)
Supplement: S2 File — (DOCX) [file pone.0269061.s006.docx]

*Prediction equations in three anatomical planes: Examination of difficulties in modeling joint variables (i.e., angles, moment, power)*

Fitting a model assumes that there is a typical behavior that can be modeled and that is comparable between participants and slopes. However, for several parameters in the frontal and transverse planes, the normalized stride cycles showed small change in their value range during the cycle, thus making it difficult to fit a Fourier series that describes them well. Most of the sources of errors in fit are due to the first coefficients of the polynomial regression, which are not well fitted (**Fig S2A**). Thus, while the final fit follows the averaged signal, it has an offset of each slope (**Fig S2B**). This results in low adjusted R^2^ values, even though the initial fit for each slope was very good individually.
