# Supplementary material for: Parametric equations to study and predict lower-limb joint kinematics and kinetics during human walking and slow running on slopes
Source: PLoS One. 2022 Aug 4;17(8):e0269061. doi: 10.1371/journal.pone.0269061 (PMC9352080; doi:10.1371/journal.pone.0269061)
Supplement: S3 File — (DOCX) [file pone.0269061.s007.docx]

*Content of supplementary data*

This folder contain data from all the participants both the ones that the equations where developed based on them (Train Data) and the ones used for testing the equations (Test Data) Content:

Data files

• Train Data – Data used for model training

o A folder for each anatomical plane

▪ ALL_NORM_DATA file – normalized gait cycle, averaged on all participants, on each surface gradient, with separate Excel sheet for each lower limb joint variable.

▪ A separate file for each lower limb joint variable. Normalized gait cycle for each participant, for joint variable (e.g., ankle angel and sagittal planeANG_data_ANK) with separate Excel sheet for each surface gradient.

• Test Data – Data used for model testing

o Subfolders as in Train Data folder

Parametric equations files

• Coefs – model coefficients.

o A folder for each anatomical plane

▪ A folder for each activity type (Walk/Run)

• A file for each lower limb joint variable.

o Female-Male models - coefficients for separate models for male and female participants. Sub folders with the same structure as described above.

• Model_coefs - .mat file containing series sizes for each lower limb joint variable at each anatomical plane.

• paper_equations_walk.m / paper_equations_run.m – MATLAB codes for generating the average time series of predicted lower-limb joint variables while walking or running at any gradient within the trained ranges. These codes use coefficients from the files listed above.

o These codes can be used to generate averaged time series for female and male separately using the coefficients provided, just set the right path in ‘CSV_path’ variable. On line 46 in the script, change (for female model):

CSV_path = ['Coefs\' anat_plane '\RUN\'];

To:

CSV_path = ['Coefs\Female-Male models\Female' anat_plane '\RUN\'];

o If one wishes to plot the standard deviation of the averaged normalized gait cycles as well as the fit, it can be calculated from the data provided in the Train/Test Data folders.
